# Four Years of Disease-Free Survival After Conservative Treatment of Subglottic Adenoid Cystic Carcinoma

**DOI:** 10.7759/cureus.28377

**Published:** 2022-08-25

**Authors:** Chrysoula Vardaxi, Antonios Skalias, Paraskevi Karamitsou, Evropi Forozidou, Alexandros Poutoglidis

**Affiliations:** 1 Department of Otorhinolaryngology-Head and Neck Surgery, “G. Papanikolaou” General Hospital, Thessaloniki, GRC

**Keywords:** oncology, chemoradiation, salivary gland tumors, larynx, adenoid cystic carcinoma

## Abstract

Adenoid cystic carcinoma (ACC) is a rare malignancy of the larynx. Surgical excision seems to be the preferred treatment modality; however, a paucity of high-evidence suggestions due to the small number of cases have been noted. Here we present the case of a 35-year-old woman with subglottic ACC who denied laryngectomy and opted for concurrent chemotherapy and radiotherapy. She remains disease-free four years later. This case illustrates that concurrent chemoradiation, instead of laryngectomy, should be considered in selected cases.

## Introduction

Squamous cell carcinoma is the most common neoplasm of the larynx [1]. While adenoid cystic carcinomas (ACCs) are the most common malignant tumors of the minor salivary glands [2], only a small minority (<1%) affects the larynx, because of the low density of submucosal salivary glands in this area [3]. Other uncommon locations in the head and neck area have been reported as well [4]. Macroscopically, the tumor is located in the submucosal layer and may often be accompanied by superficial ulcerations. Histologically, ACCs are divided into three main subtypes: cribriform, tubular, and solid. This classification is based on the cellular architecture and the different histological patterns [3,5].

Laryngeal ACC develops slowly and the symptoms tend to appear late [3]. The accurate etiology of laryngeal ACCs is still unknown, and smoking is not a proven risk factor [3]. Imaging is essential to define tumor size, local infiltration, perineural invasion, and distant metastases. Both computed tomography (CT) and magnetic resonance imaging (MRI) can sufficiently assess the location of the tumor and any extra-luminal extensions or metastases. MRI is the most sensitive modality to evaluate the perineural invasion [6]. Finally, conclusive diagnostic evidence is provided only by the histopathologic examination after a biopsy or total excision of the neoplasm [5,6]. According to current literature, surgical excision is the gold standard modality for the treatment of ACC independently of its location.

In this paper, we report a case of a laryngeal ACC which was treated without surgery and remains disease-free after four years.

## Case presentation

A 35-year-old woman presented to our emergency ENT department with the chief complaint of progressive dyspnea and inspiratory stridor over the past three months. She reported hoarseness and mild dysphagia for two years but no otalgia or weight loss. She has consumed alcohol socially and smoked 20 cigarettes per day for 15 years. She is otherwise healthy and employed as a babysitter.

**Figure 1:**
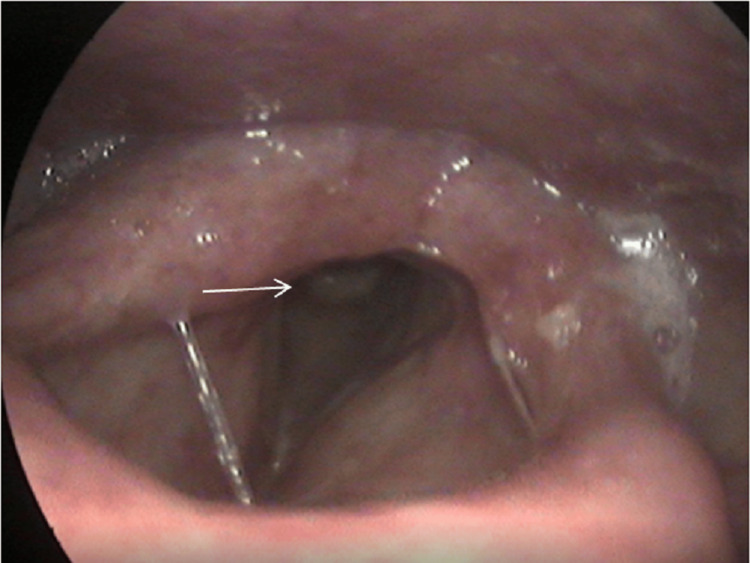
Endoscopic image at the time of initial presentation reveals a subglottic mass (white arrow)

On physical examination, a subglottic tumor was detected through rigid endoscopic laryngoscopy. The mass was smooth, not ulcerated or friable, located below the vocal folds, and narrowing the airway (Figure 1). Vocal fold mobility was not impaired and glottic closure was adequate. Neck palpation did not reveal any enlarged or suspicious lymph nodes.

The patient had undergone a cervical MRI and chest CT in another hospital three days before her referral to the emergency department of our hospital. These studies revealed a 1.6x1.8x1.3 cm subglottic mass without any involvement of laryngeal cartilages. Neither adenopathy nor distant metastases were identified.

**Figure 2:**
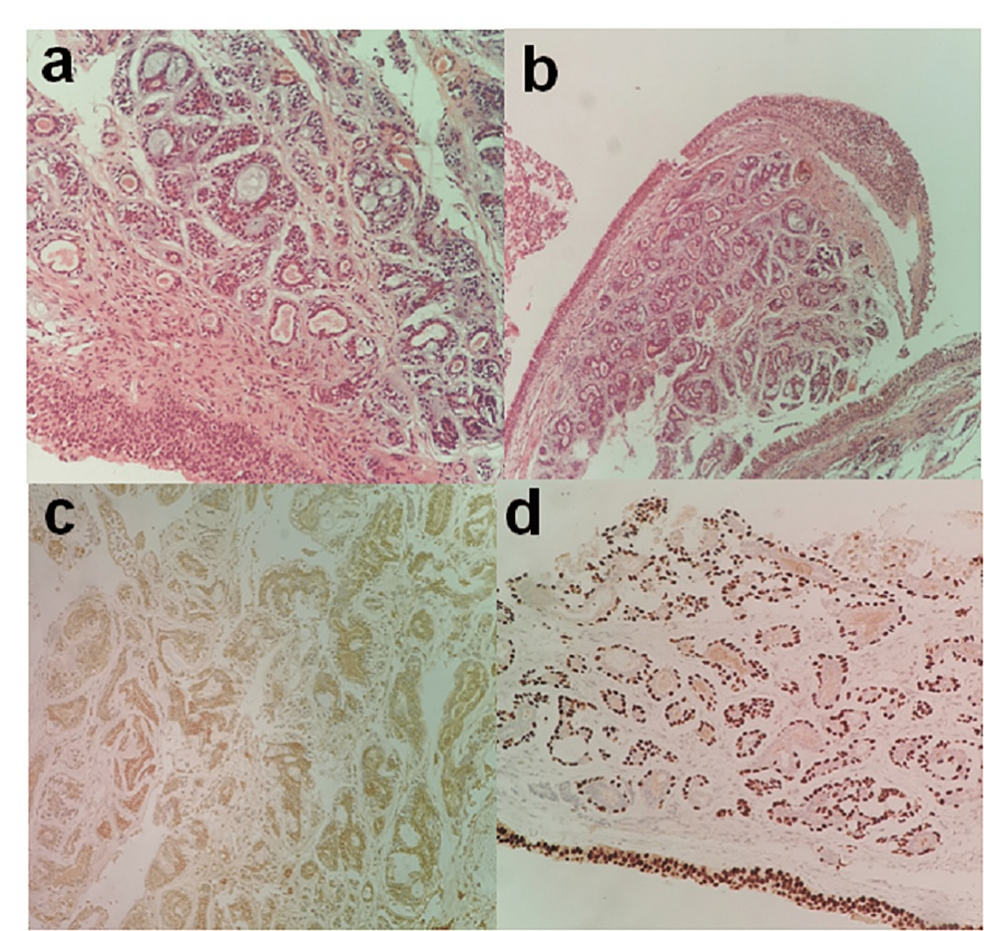
(a) Evident biphasic nature with ductal cells towards the luminal surface and myoepithelial externally (H&E, 20X), (b) Tumor cells organized in tubular and cribriform formations dispersed inside lamina propria (H&E, 10X), (c) Membranous staining highlighting ductal cells (immunohistochemical staining for c-Kit, 20X), (d) Nuclei of myoepithelial cells (P63, 20X) H&E: hematoxylin and eosin staining

An emergency tracheotomy was performed under local anesthesia to secure the airway because of the obstructive nature of the neoplasm. A panendoscopy for assessment of the upper aerodigestive tract was performed. Panendoscopy included biopsies from every site of the upper aerodigestive tract suspicious for malignancy (nasopharynx, soft palate, tongue base, tonsils, larynx, and esophagus). Biopsies revealed a subglottic laryngeal adenocystic carcinoma of a cribriform pattern (Figure 2).

The patient was informed about the pathology results and a total laryngectomy was suggested as the treatment of choice. Every complication of the operation and every impairment in the quality of life was meticulously explained. The patient denied the operation and opted for concurrent chemotherapy and radiotherapy to avoid a permanent tracheostomy. Thirty-three courses of radiation with a total of 66 Gray and seven weekly cycles of platinum-based chemotherapy were conducted. Lymph nodes of the neck were excluded from the radiation field because of the histologic nature of the tumor. Two months after the end of the treatment the tracheostomy tube was removed uneventfully.

The patient was informed that a lifelong follow-up is mandatory and that in case of a local recurrence a salvage laryngectomy will probably be the only available modality. Lifelong follow-up includes at least a visit per year even after 10 years of treatment due to the tendency of neoplasm to give late metastasis. A new MRI was performed 18 months after the treatment, which did not reveal any abnormal imaging findings. Four years later and to date the patient remains disease-free with only the minor complaint of dryness in the oral cavity. A flexible laryngoscopy was performed every three months and yearly MRIs did not reveal any recurrence or metastasis.

## Discussion

ACC is a rare malignancy of the larynx, with a mean age of diagnosis between the fifth and sixth decade of life and with no clear prevalence between genders [5]. Subglottic ACCs account for 60% of the cases and tend to extend in the trachea. On the other hand, supraglottic involvement is identified in 35% of cases, and the glottic region is affected extremely rarely [3].

There are no specific guidelines or consensus for the treatment of laryngeal ACCs. The literature lacks high-evidence suggestions grounded on organized cohorts or trials, because of the rarity of the neoplasm. The proposals found in the literature are based typically on case reports or small case series [7-10]. Surgical resection seems to be the selected treatment modality and is often accompanied by postoperative radiotherapy. Operative treatment includes a total or a partial laryngectomy based on the tumor location [3,5]. Neck dissection or elective radiation is not required, because lymphatic spreading is extremely rare [5,11]. Generally, surgery seems to offer a better survival rate in patients. Adjuvant radiotherapy is mostly indicated in positive or close resection margins, lymphovascular invasion, perineural invasion, and positive nodal status [3]. Radiotherapy is not suggested as monotherapy. The use of fast neutron or proton radiotherapy and carbon ion radiotherapy has been tested on ACCs, resulting in a satisfactory outcome for those that cannot be operated, [12]. Moreover, the value of chemotherapy is still under discussion, although it is recommended by some authors for advanced or recurrent cases. Patients diagnosed with laryngeal ACCs require long-term follow-up, independently of the therapy [5]. It is remarkable that one-fifth of patients have a recurrence 10 years after the original treatment [12]. The five-year survival rate may vary from 43% to 75%, as far as can be estimated by the existing literature [3,12].

A systematic review reported only four cases treated with chemoradiation for organ preservation considerations [11]. Half of the above patients were free of disease at follow-up, without radiologic or clinical evidence of recurrence, four years after the original diagnosis. Two out of four had metastases about 18 and 54 months after treatment. The longest survival reported among those patients was 112 months [11].

Additionally, distant metastases leading to late recurrence are not uncommon [13,14]. Radical surgical treatment does not affect those cases. As a result, the objective of organ-preserving approaches should be carefully considered.

## Conclusions

The role of concurrent chemoradiation for laryngeal ACCs, however controversial, has the huge advantage of larynx preservation. Even though surgical resection is the treatment modality of choice, late distant metastases occur often. The successful course of our case so far suggests that concurrent chemoradiation may be equal or even superior to surgical resection in selected cases, especially when combining considerations of patients’ quality of life and survival rate. As more cases are discovered and reported, meta-analyses may serve to uncover more details regarding this treatment dilemma. Furthermore, there is a necessity for large prospective studies to clarify the advantages and disadvantages of every method in an evidence-based fashion.
